# Assessment of the Usefulness and Reliability of YouTube Videos on Postmortem Procedures

**DOI:** 10.7759/cureus.79412

**Published:** 2025-02-21

**Authors:** Anamika Nath, Anupam Datta

**Affiliations:** 1 Forensic Medicine, Tezpur Medical College and Hospital, Tezpur, IND; 2 Forensic Medicine, Agartala Government Medical College and Govind Ballabh Pant Hospital (GBP) Hospital, Agartala, IND

**Keywords:** educational videos, medical content, postmortem procedures, reliability, usefulness, youtube™

## Abstract

Introduction: The increasing reliance on digital platforms for educational purposes has resulted in the widespread availability of medical content, including videos on postmortem procedures on YouTube (Google Inc., Mountain View, CA). The present study analyzed the significance of YouTube videos on postmortem procedures to use as an effective educational tool.

Methods: A comprehensive search of YouTube videos on postmortem procedures was conducted using the keyword "postmortem" on a specific date. Videos were included if they were in English, provided content directly related to postmortem procedures, and included audio narration or explanatory text. Data collection and analysis involved evaluating video quality, engagement metrics, and content accuracy using various scales and statistical tests.

Results: The study analyzed 50 YouTube videos on postmortem procedures, finding that most (n=32, 64%) were of low usefulness, and the majority had low reliability scores. Engagement metrics showed a mean view ratio of 1,500 views per day and an average like ratio of 5.6 but were not predictive of video quality.

Conclusion: The majority of YouTube videos on postmortem procedures lack educational value and reliability, emphasizing the need for credible organizations to create high-quality digital resources.

## Introduction

Digital platforms like YouTube (Google Inc., Mountain View, CA) have revolutionized access to medical knowledge, providing information to both the public and healthcare professionals. Among these, videos related to postmortem procedures have garnered significant attention. They serve as educational tools for medical students and a source of general information for the public. Despite its potential, the platform faces criticism for inconsistent content quality, often influenced by the expertise of content creators and their intent [1,2]. The accurate representation of postmortem procedures is crucial for dispelling myths and aiding understanding. Previous studies have documented the variability in the quality of online medical content, highlighting the need for systematic evaluation [3,4]. The aims and objectives of this study are to comprehensively evaluate the quality of YouTube videos related to postmortem procedures. Specifically, the study seeks to assess the usefulness and reliability of these videos, examining their educational value and credibility. Additionally, the study aims to investigate the relationship between engagement metrics, such as views and likes, and the educational value of the videos. Ultimately, the study aims to identify gaps in the quality of digital educational content on postmortem procedures, providing insights for improvement and informing the development of high-quality educational resources.

## Materials and methods

Study design and search strategy

This cross-sectional study systematically evaluated YouTube videos on postmortem procedures. A comprehensive search was conducted using the keyword "postmortem" on June 10, 2024. Videos were retrieved based on relevance, and automated filters were used to exclude content unrelated to medical postmortem procedures. To enhance reproducibility, the search was conducted in incognito mode to avoid algorithmic bias. The videos were categorized based on the individual or organization that uploaded the video on YouTube, like medical professionals, allied health professionals, helpers of health professionals, and other enthusiasts.

Study settings

Since this research did not involve any human participants or their data but only freely available data on the internet regarding postmortem videos, it was not conducted in any institution specifically, but the authors met at a common place for the selection of videos.

Inclusion criteria

Videos were included if they were in English, provided content directly related to postmortem procedures, and included audio narration or explanatory text.

Exclusion criteria

Duplicated videos, content in languages other than English, and silent or contextually irrelevant videos were excluded from the study.

Data collection and metrics

Quantitative data, including video duration, number of views, likes, dislikes, and upload dates, were collected. Engagement metrics were further analyzed, including the view ratio (views per day) and like ratio (likes to dislikes) as a part of engagement metrics, and interaction index and video power index (VPI). 

The various matrix and indexes were computed as follows: $View Ratio = \frac{\text{Number of views}}{\text{Number of days posted}}$

$Like Ratio = \frac{\text{Number of likes}}{\text{Number of likes} + \text{Number of dislikes}}$[5]

$Interaction Index = \left( \frac{\text{Number of likes} - \text{Number of dislikes}}{\text{Number of likes}} \right) \times 100$

$Video Power Index = \frac{\text{Like Ratio} \times \text{View Ratio}}{100}$ [6]

Engagement metrics determine the level of interaction between the audience and the video publisher. The interaction index indicates the level of engagement the audiences have with the uploader’s content, with high values indicating higher engagement. The VPI is a measure of the popularity of the video; the higher the VPI, the more popular the video is among users.

The different scales used were the Global Quality Assessment Scale (GQS) and the Modified DISCERN (mDISCERN) tool [7,8]. The scales and their description are elaborated in Table 1. 

**Table 1:** Description of the scales used in the study

Serial No.	Name of the scale	Description of the scale	Significance
1.	Global Quality Assessment Scale (GQS) [7]	The Global Quality Scale (GQS) serves as an instrument for evaluating the quality of information presented in videos, including those available on YouTube. This scale can be applied to assess the quality of videos across various subjects.	This tool rated video quality on a five-point Likert scale, focusing on audiovisual content and comprehensiveness. A score of one on the GQS indicates that a video is of poor quality with a poor flow of information and is of very little use to viewers, whereas, a score of five indicates a video is of excellent quality with a good flow of information that is very useful for viewers.
2.	Modified DISCERN Tool (mDISCERN) [8]	The Modified DISCERN Tool denotes a streamlined adaptation of the original DISCERN tool, designed to assess the reliability and quality of health information, especially from online platforms such as YouTube videos. This version emphasizes essential elements of credibility and facilitates a more rapid evaluation through a reduced set of questions.	Reliability is assessed using this validated instrument, with higher scores reflecting greater trustworthiness. It is usually scored on a five-point scale where higher scores indicate greater reliability.1 point for every "yes", 0 points for every "no". i. Are the aims clear and achieved? ii. Are reliable sources of information used? (i.e., the publication cited, the speaker is a specialist in that particular discipline) iii. Is the information presented both balanced and unbiased? iv. Are additional sources of information listed for patient reference? v. Are areas of uncertainty mentioned?

For content scoring six broad areas were looked into, each consisting of two items, which can be found in Table 2. 

**Table 2:** Description of the criteria for content scoring

Serial No.	Name of the content	Criteria of the content	Scoring system
1.	Post-mortem procedure overview	1. Explains the purpose and steps of the post-mortem procedure; 2. Differentiates between types of autopsies (forensic, clinical)	No information: 0; Partial information: 1; Complete information: 2
2.	Cause of death identification	1. Describes the methods used to determine the cause of death; 2. Highlights common causes of death and verification techniques	No information: 0; Partial information: 1; Complete information: 2
3.	Forensic techniques used	1. Demonstrates the use of forensic tools and explains their purpose; 2. Accurate portrayal of toxicology, histopathology, and other techniques	No information: 0; Partial information: 1; Complete information: 2
4.	Ethical and legal considerations	1. Discusses ethical issues such as privacy and consent for sharing post-mortem footage; 2. Adheres to legal guidelines for recording and sharing autopsy videos	No information: 0; Partial information: 1; Complete information: 2
5.	Anatomical and pathological details	1. Provides detailed anatomical and pathological insights during the autopsy; 2. Educates viewers on identifying abnormalities or causes of death	No information: 0; Partial information: 1; Complete information: 2
6.	Post-mortem challenges and follow-up actions	1. Identifies potential complications and errors during autopsy procedures; 2. Suggests follow-up actions, legal implications, and further forensic testing if needed	No information: 0; Partial information: 1; Complete information: 2

For video evaluation, the sum of the GQS and the content score was estimated to evaluate the usefulness score of a video. The usefulness scores could range anywhere between one and 17, as described in Table 3. 

**Table 3:** Range score for usefulness for video evaluation

Usefulness score for video evaluation	Range category
1 - 5	Low
6 - 11	Medium
12 - 17	High

The scores given by two researchers were averaged to give an overall score, which was then used in the analysis. Two independent reviewers assessed each video to ensure consistency in scoring. The inter-class correlation coefficient (ICC) was calculated for the GQS, usefulness score, and mDISCERN score, with values >0.75 indicating strong agreement.

Statistical analysis

Data analysis was performed using IBM SPSS Statistics software, version 27.0 (IBM Corp., Armonk, NY) [9]. The Shapiro-Wilk test was used to check the normality of the data. Non-parametric tests, such as the Kruskal-Wallis test, were employed to check whether differences existed in video characteristics, engagement metrics, usefulness, and mDISCERN scores based on the source of videos. Categorical variables were presented with frequency and percentages. Non-normally distributed continuous variables were presented with medians and interquartile ranges (IQRs). Multiple linear regression analysis was conducted with the usefulness score as the dependent variable and video engagement metrics and mDISCERN score as independent variables.

Ethical consideration

These videos were sourced from freely available public domain accessible to all. No identifiers were present; hence, the questions of breach of identity, confidentiality, and other ethical aspects do not arise.

## Results

Video characteristics and engagement metrics

**Figure 1:**
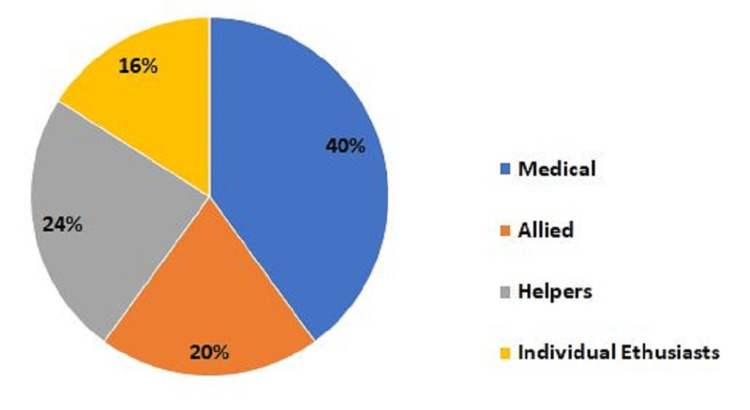
A pie chart showing the source of the videos This image has been created by the authors.

Of the 70 videos retrieved, 50 met the inclusion criteria, with a total duration of 21.7 hours and a total of over 23 million views. Videos were predominantly uploaded by medical and allied health practitioners (n=30, 60%) (Figure 1).

Engagement metrics showed a mean view ratio of 1,500 views per day and an average like ratio of 5.6. The ICC values were 0.81, 0.84, and 0.76 for the GQS, usefulness score, and mDISCERN score, respectively, indicating high inter-rater reliability. Video characteristics analyzed their duration, views, likes, and engagement levels (Table 4). The median video duration was 6.5 minutes, with an average view ratio of 1,500 views per day. The like ratio was 0.91, indicating moderate audience appreciation. 

**Table 4:** Characteristics of postmortem videos

Variables	Median (min, max)
Posted days	789 (60, 4032)
Duration in minutes	6.5 (0.5, 48.45)
Views in numbers	1120 (20, 520000)
View ratio	0.79 (0.01, 150.00)
Likes in numbers	12 (0, 2100)
Dislikes in numbers	1 (0, 150)
Like ratio	0.91 (0.0, 1)
Interaction index	0.43 (-0.20, 10.50)
Video power index	0.06 (0.0, 1.50)
Usefulness score	6 (1, 15)
Modified DISCERN (mDISCERN) tool score	1.8 (0.5, 3.5)

Usefulness and reliability

Usefulness Scores

**Figure 2:**
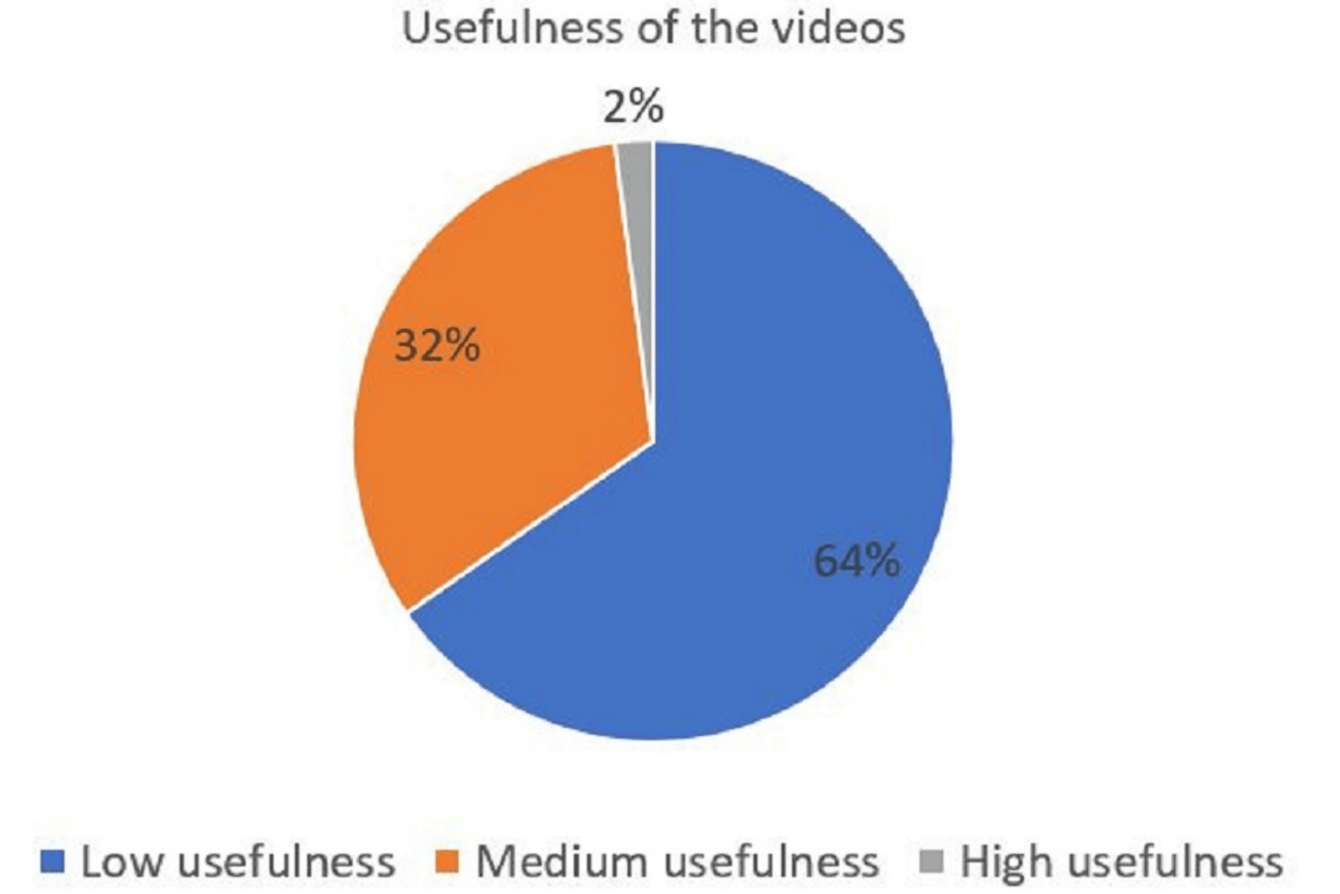
Usefulness of the videos This image has been created by the authors.

Most videos (n=32, 64%) were categorized as low usefulness (Figure 2).

The mDISCERN Scores

Approximately 74.2% of videos scored two or below, reflecting low reliability.

Content Score

The content analysis of YouTube videos on postmortem procedures revealed significant gaps in educational quality, as seen in Table 5. Only 30% of videos provided complete details on the post-mortem procedure overview, while 40% contained partial information. In terms of cause of death identification, merely 20% offered comprehensive content, with 50% presenting partial details. The use of forensic techniques was inadequately covered, as 48% of videos lacked proper explanations. Ethical considerations were the most neglected aspect, with 70% of videos failing to discuss legal and ethical issues. Similarly, anatomical and pathological details were only fully covered in 24% of videos, whereas 36% lacked relevant insights. Additionally, 60% of videos did not address post-mortem challenges and follow-up actions, highlighting the need for more structured and informative content in forensic education.

**Table 5:** Content score for post-mortem videos

Content category	Complete information n(%)	Partial information n(%)	No information n(%)
Post-mortem procedure overview	15 (30%)	20 (40%)	15 (30%)
Cause of death identification	10 (20%)	25 (50%)	15 (30%)
Forensic techniques used	8 (16%)	18 (36%)	24 (48%)
Legal and ethical considerations	5 (10%)	10 (20%)	35 (70%)
Anatomical and pathological details	12 (24%)	20 (40%)	18 (36%)
Post-mortem challenges and follow-up actions	6 (12%)	14 (28%)	30 (60%)

Statistical Insights

The Shapiro-Wilk test indicated that the data were not normally distributed for one or more variables. Hence, the Kruskal-Wallis test was done, which showed a positive correlation was observed between mDISCERN scores and usefulness scores (H = 14.160, p < 0.001), while engagement metrics were not predictive of video quality.

Table 6 shows the multiple regression model using usefulness as the dependent variable. The statistical model examined factors influencing usefulness scores. mDISCERN scores significantly predicted usefulness (p < 0.001), confirming that video reliability strongly determines educational value. However, engagement metrics like likes, views, and interaction index were not predictive of video usefulness.

**Table 6:** Multiple linear regression with usefulness as the dependent variable Regression coefficient (B) along with its 95% confidence interval (CI); SE: standard error; mDISCERN: modified DISCERN; VPI: video power index

	B(95% CI)	SE	Significance
Like ratio	0.04 (-1.18 -1.27)	0.61	0.94
View ratio	-0.02 (-0.1 -0.07)	0.04	0.69
Interaction index	0.18 (-0.03-0.39)	0.1	0.09
VPI	2.48 (-8.59-13.555)	5.59	0.65
mDISCERN score	2.08 (1.31-2.85)	0.38	<0.001

The mDISCERN scores significantly predict usefulness, while engagement metrics do not, underscoring that reliability is a stronger determinant of educational value than audience interaction. Variations in reliability and usefulness across different uploader categories were checked. Videos uploaded by medical professionals had higher usefulness and reliability scores compared to those from individual enthusiasts. The median usefulness score for medical professionals was 7.25, whereas individual enthusiasts’ videos had a median score of four. Similarly, the mDISCERN scores were higher for medically sourced videos, confirming their credibility over user-generated content, as seen in Table 7.

**Table 7:** Reliability and usefulness of postmortem videos on YouTube based on source of videos (median (IQR)) IQR: Interquartile range; mDISCERN: modified DISCERN; VPI: video power index; Significance: *p < 0.05: **p <0.001

Characteristics	Medical (Median (IQR))	Allied health practitioners (Median (IQR))	Helpers (Median (IQR))	Individual enthusiasts (Median (IQR))	H-value
Duration (minutes)	2.15 (1.38, 4.23)	4.01 (2.52, 6.08)	5.51 (3.12, 8.14)	5.51 (4.22,9.17)	615.243^**^
Number of days posted	1289 (498, 2569)	792 (377, 1756)	1542 (812, 2249)	2415 (1638, 3393)	6.489^*^
Number of views	701.5 (94.714, 959.5)	467 (50, 3409)	195 (75, 1125)	78 (15,113, 5050)	1.417
Likes	11.5 (0.0, 94)	2(0.0, 12)	4 (0.0, 22)	2 (0.0, 459)	2.016
Dislikes	0.0 (0.0, 5.50)	0.0 (0.0, 1)	0.0 (0.0, 4)	0.0 (0.0, 77)	2.994
Like ratio	0.93 (0.0, 1)	0.95 (0.0, 1)	0.9 (0.5, 1)	0.89 (0.0, 1)	0.426
View ratio	0.95 (0.0, 0.11)	0.63 (0.13, 3.34)	0.4 (0.07, 523)	0.06 (0.01, 69.29)	1.422
VPI	0.01 (0.0, 0.11)	0.0 (0.0, 0.02)	0.0 (0.0, 0.09)	0.0 (0.0, 0.6)	3.150
Interaction index	0.49 (0.0, 1.45)	0.32 (0.0, 1.11)	0.39 (0.0, 1.67)	0.23 (0.0, 1.33)	0.316
Usefulness score	7.25 (5.5, 9.12)	4.4 (3, 5.5)	5.75 (4. 12)	4 (3.5, 10.5)	26.707^**^
mDISCERN score	2 (2, 2.5)	2 (1.5, 2)	2 (1.5, 2)	2 (1.5, 2)	1.973

## Discussion

This study comprehensively assessed the educational value and reliability of YouTube videos on postmortem procedures. The findings reveal a critical gap in the quality of available content, with a majority of videos rated as low in usefulness and reliability. These deficiencies are particularly concerning given the increasing reliance on digital platforms for medical education and public awareness. The strong correlation between reliability (as measured by mDISCERN scores) and usefulness underscores the importance of evidence-based, expert-led content in enhancing the educational potential of online resources. However, the lack of significant differences in engagement metrics across usefulness categories highlights the inadequacy of user interactions, such as likes and views, as indicators of content quality. This further reinforces the need for standardized evaluation frameworks to guide users in identifying credible and valuable resources. The high proportion of low-usefulness videos underscores the variability in content quality. These findings align with earlier studies indicating that non-expert creators often produce suboptimal educational material [7]. The strong positive correlation between mDISCERN and usefulness scores demonstrates that reliability is a cornerstone of educational value. This reinforces the importance of evidence-based, well-researched content in enhancing learning outcomes.

Despite the intuitive appeal of using likes, views, and other metrics as proxies for quality, this study confirms their unreliability. Engagement is often driven by factors like entertainment value rather than educational merit, necessitating alternative evaluation frameworks for medical content. Unlike previous research reporting a dominance of user-generated content, this study observed a significant contribution from allied health practitioners [8]. This shift may be attributed to increased professional participation or variations in video accessibility algorithms. Many videos available freely lack peer review or expert validation, which raises concerns about their accuracy and reliability as medical education resources. Drozd et al. highlighted the lack of standardized evaluation methods for medical YouTube content, emphasizing that popularity does not necessarily correlate with accuracy and educational value [10]. Similarly, Azer et al. found that only a small proportion of videos on cardiovascular and respiratory examinations were of high educational quality, with many containing misleading or incomplete information [11]. These studies reinforce our conclusion that content reliability is a more crucial determinant of educational value than engagement metrics such as views and likes. Additional studies have identified similar trends across various medical disciplines. Singh et al. investigated YouTube videos on rheumatoid arthritis and found that a majority contained biased or misleading information [1]. Knösel and Jung observed that orthodontic videos often lacked scientific rigor and were more promotional in nature. These studies demonstrate that the challenge of unreliable content is not limited to postmortem procedures but extends across multiple medical fields [12]. Furthermore, our study observed that videos uploaded by medical professionals had higher reliability and usefulness scores compared to those created by enthusiasts or allied health professionals. This is consistent with Leong et al., who found that diabetes-related videos produced by healthcare institutions were more accurate and informative than those created by non-experts [3]. Sahin et al. also noted that colorectal cancer videos made by medical organizations were significantly more reliable than patient-generated content. These findings highlight the importance of expert-led content creation in ensuring the accuracy of medical information on YouTube [4].

Our study further supports the argument that engagement metrics are poor indicators of educational value. While some videos garnered high viewership and engagement, their content often lacked scientific rigor. Given the widespread accessibility of YouTube as an educational tool, it is imperative for medical associations and academic institutions to take a more active role in content production. Standardized guidelines for video creation, peer review processes, and labeling verified medical content can help bridge the gap between accessibility and reliability. Previous recommendations by Charnock et al. through the DISCERN tool have underscored the need for critical appraisal of online health information, which remains relevant in the current digital age [8]. A recent systematic review by Kocyigit BF et al. further emphasizes the limitations of YouTube as a reliable source of medical education [13]. Their analysis of orthopedic surgery videos found that most content lacked comprehensive explanations, with only a few videos created by recognized experts. This is in agreement with our findings, highlighting the pervasive issue of suboptimal quality across various medical disciplines. Moreover, studies by Syed-Abdul S et al. have shown medical misinformation is vast [14]. Also, it has been demonstrated that medical misinformation is widespread on YouTube, necessitating stringent quality control measures (conference souvenir: Gabarron E, Larbi D, Denecke K, Årsand E, Wynn R. What Do We Know About the Impact of Online Misinformation on Public Health? A Systematic Review. Annual Hawaii International Conference on System Sciences; January 8 - 11, 2019). These studies further validate our argument that medical professionals and regulatory bodies must take active steps to improve content quality on digital platforms. The growing use of artificial intelligence (AI) in content moderation offers a potential solution. As suggested by Tangcharoensathien et al., AI-driven verification processes could enhance the accuracy of online medical videos, flagging misinformation and promoting credible content [15]. Such interventions could help bridge the gap between public access and scientific reliability in digital medical education.

These findings underscore the urgent need for authoritative bodies, such as medical associations and academic institutions, to prioritize the creation of high-quality educational videos. Such initiatives could standardize online learning resources and reduce misinformation, particularly on sensitive topics like postmortem procedures. This study comprehensively assessed the educational value and reliability of YouTube videos on postmortem procedures. The findings reveal a critical gap in the quality of available content, with a majority of videos rated as low in usefulness and reliability. These deficiencies are particularly concerning given the increasing reliance on digital platforms for medical education and public awareness. Though there are no standardized guidelines for sharing postmortem videos online, the authors recommend establishing a social media policy that outlines the procedure for sharing sensitive and personal information globally. There should also be age restrictions for viewing sensitive content like postmortem videos so that minors cannot access the same. Protection of the confidentiality of the deceased along with the dignity of the dead must be maintained throughout.

Limitations

The limitations were the sample size, which was relatively small, and the study only evaluated videos related to postmortem procedures. Secondly, the evaluation tools used may not have captured all aspects of video quality. The study did not evaluate the impact of the videos on viewer knowledge or behavior. This study evaluated only English-language videos, which may not represent global trends. The custom usefulness scoring system, while reliable, could benefit from further validation. Finally, the cross-sectional design limits the ability to assess trends over time.

## Conclusions

The majority of YouTube videos covering postmortem procedures are of poor educational value and reliability. To address this shortcoming, credible organizations must create high-quality digital resources, ensuring that accessible and accurate medical education reaches diverse audiences. Future studies should investigate changes over time, incorporate multilingual content, and evaluate the impact of interventions by authoritative bodies in enhancing online medical education. Addressing these issues comprehensively can help bridge the gap between accessible information and reliable education, ensuring that platforms like YouTube serve as trustworthy sources for all viewers. YouTube videos on postmortem procedures can serve as a valuable resource for medical professionals, students, and researchers. However, it is essential to evaluate the credibility and reliability of these videos by examining the qualifications of the content creator and considering feedback from other viewers. By critically assessing the material, viewers can maximize the educational potential of YouTube as a tool for learning about postmortem procedures.
